# One-bead one-compound combinatorial library derived targeting ligands for detection and treatment of oral squamous cancer

**DOI:** 10.18632/oncotarget.27189

**Published:** 2019-09-10

**Authors:** Fan Yang, Wenwu Xiao, Yanlei Liu, Ruiwu Liu, Randall Kramer, Xiaocen Li, Yousif Ajena, Christopher M. Baehr, Tatu Rojalin, Hongyong Zhang, Kit S. Lam

**Affiliations:** ^1^ Department of Oral Medicine, Infection and Immunity, Harvard School of Dental Medicine, Boston, MA, USA; ^2^ Department of Biochemistry and Molecular Medicine, University of California, Davis, CA, USA; ^3^ Department of Pathology, University of California Davis Medical Center, Sacramento, CA, USA; ^4^ Department of Cell and Tissue Biology, University of California, San Francisco, CA, USA

**Keywords:** oral squamous cancer, α3 integrin, optical imaging, cancer-targeting peptide, orthotopic xenograft model

## Abstract

Oral squamous cancers (OSC) are hallmarked by poor prognosis, delayed clinical detection, and a lack of defined, characteristic biomarkers. By screening combinatorial one-bead one-compound (OBOC) peptide libraries against oral squamous cancer cell lines, two cyclic peptide ligands, LLY12 and LLY13 were previously identified. These ligands are capable of specific binding to the oral cancer cell lines (MOK-101, HSC-3, SCC-4 and SCC-10a) but not non-cancerous keratinocytes, leukocytes, fibroblast, and endothelial cells. These two peptides were synthesized and evaluated for their binding property, cytotoxicity and cell permeability. *In vitro* studies indicate that both LLY12 and LLY13 were able to bind to oral cancer cells with high specificity but did not show any cytotoxicity against human keratinocytes. Biotinylated LLY13, in complex with streptavidin-alexa488 was taken up by live oral cancer cells, thus rendering it as an excellent candidate vehicle for efficient delivery of drug loaded-nanoparticles. *In vivo* and *ex vivo* near infra-red fluorescence imaging studies confirmed the *in vivo* targeting efficiency and specificity of LLY13 in oral cancer orthotopic murine xenograft model. *In vivo* studies also showed that LLY13 was able to accumulate in the OSC tumors and demarcate the tumor margins in orthotopic xenograft model. Together, our data supports LLY13 as a promising theranostic agent against OSC.

## INTRODUCTION

Despite the current aggressive treatment strategies with chemotherapy, radiation therapy and surgery, oral cancer remains the third most lethal cancer, accounting for 18% of all cancer deaths and a 5-year survival of less than 50% [1, 2]. Current diagnostic techniques involve light-based detection systems, fluorescent visualizations, brush cytology and biopsy with fine needle aspiration [3–5]. Unfortunately, only 35% of cases are caught in the early stages [6, 7]. To date, there are no specific biomarkers for OSC. Therefore, there is an urgent need to look for specific molecular targets of OSC to improve its early detection techniques and subsequent innovative therapies. Histopathologically, oral squamous cancer (OSC) is by far the most common cancer type of the oropharynx and oral cavity, representing more than 90% of all oral cancers [8, 9]. Oral squamous cancer of the tongue is the most common subtype of cancer diagnosed in the oral cavity comprising 25–40% of oral cancer [10, 11]. In addition, the structure of tongue which encompasses a rich lymphatic network and dense muscular tissue makes it a prime target for tumor invasion and metastasis. OSC of the tongue is thus more frequently associated with metastasis to draining lymph nodes than any other cancer of the oral cavity [12, 13].

One-bead one-compound (OBOC) combinatorial technology is a powerful approach for the identification of ligands as well as targets against live tumor cells [14–19]. OBOC combinatorial libraries can be created using synthetic chemistry to link various building blocks to form a large number of compounds, ranging from peptides to synthetic small molecules on beads, such that each bead displays an unique chemical entity [20]. In our previous studies, we selected six OBOC libraries for large-scale screening studies, in total about 3,000,000 compound beads were screened, we identified 546 OSC cell binding beads. Following the specificity filtering assay, only six compound-beads were identified from X1 focused OBOC libraries, which were capable of strong binding to different OSC cell lines but did not bind to normal human keratinocytes, endothelial cells and granulocytes [21]. X1 focused OBOC library is a cyclic peptide library with a motif (cXGXGXXc) which binds preferentially to ovarian cancer with high specificity against α3β1 integrin [22–25]. Among the six peptides identified, two demonstrated more potent binding ability and specificity on bead binding assays as well as peptide-histochemical studies. In this study, we further constructed and evaluated these two highest performing OSC-binding peptides, LLY12 (cyclic cdG (Nle) G(Hyp) Lc), and LLY13 (cyclic cdGYG (Hyp) Wc), wherein lower case letter represents D-amino acid, Nle and Hyp stand for norleucine and hydroxyproline, respectively, for their *in vitro* binding specificity and *in vivo* tumor targeting properties.

## RESULTS

### Potential cytotoxicity study of LLY12 and LLY13

**Figure 1:**
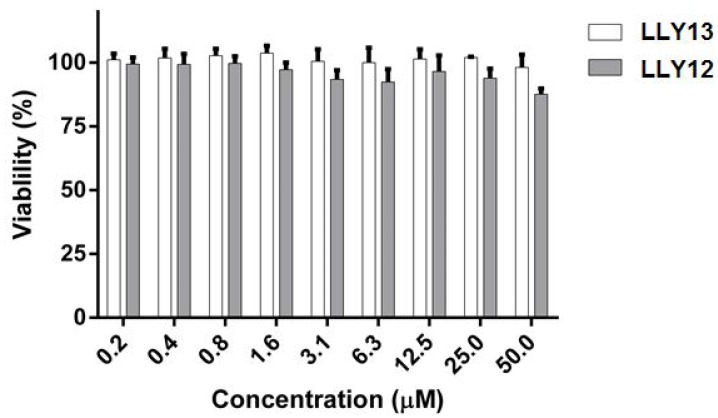
MTS assay evaluation of cytotoxicity of LLY12 and LLY13 on normal human keratinocytes (NHK). LLY12 and LLY13 were not cytotoxic to NHK cells up to the concentration of 50 μM.

Two lead peptides LLY12 and LLY13 were synthesized in soluble form. Disulfide cyclization was achieved with CLEAR-OX resin beads. The soluble peptides were purified by HPLC and the molecular weights were confirmed by mass spectrometry [17, 25]. The MTS assay was used to evaluate the potential cytotoxicity of the two purified peptides against normal human keratinocyte (NHK) cells. As shown in Figure 1, there was no obvious cytotoxicity observed for LLY12 or LLY13 up to 50 μM.

**Figure 2:**
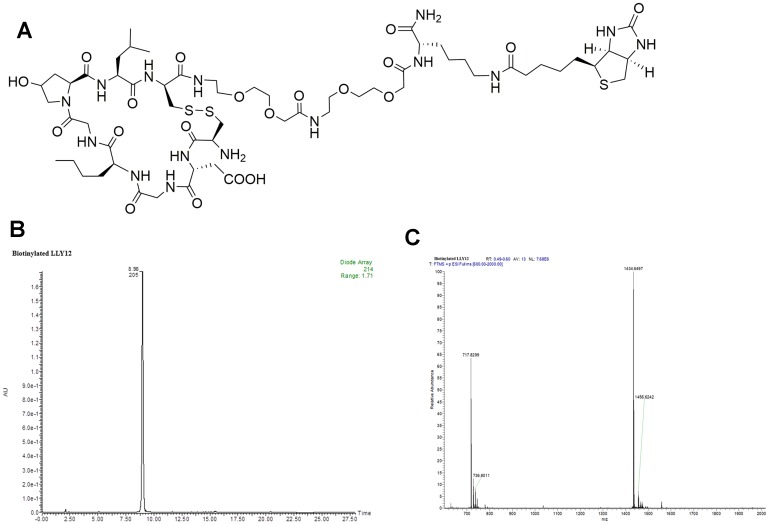
Structure and characterization of biotinylated LLY12. (**A**) The chemical structure of biotinylated LLY12, (**B**) HPLC of biotinylated LLY12, and (**C**) Orbi-trap ESI-MS: Calculated 1433.6353, Found 1434.6497 [M+H]^+^, 717.8299 [M/2+H]^+^.

### *In vitro* targeting efficacy study of LLY12 and LLY13

**Figure 3:**
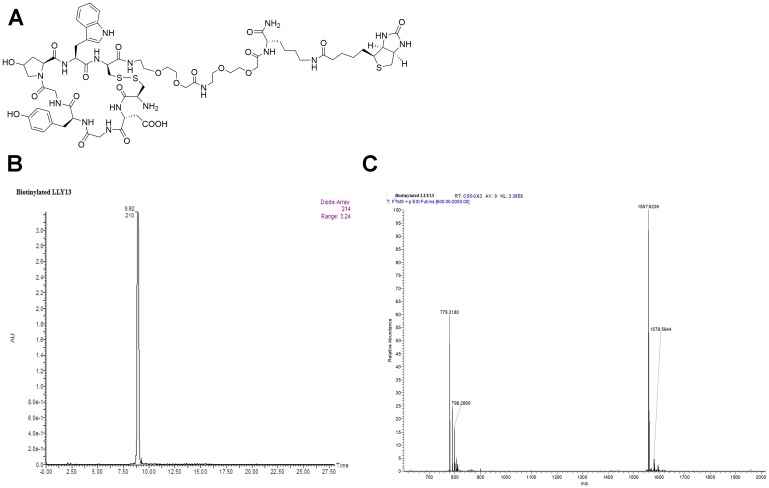
Structure and characterization of biotinylated LLY13. (**A**) The chemical structure of biotinylated LLY13, (**B**) HPLC of biotinylated LLY13, and (**C**) Orbi-trap ESI-MS of biotinylated LLY13: Calculated 1556.6098, Found 1557.6239 [M+H]^+^, 779.3180 [M/2+H]^+^.

**Figure 4:**
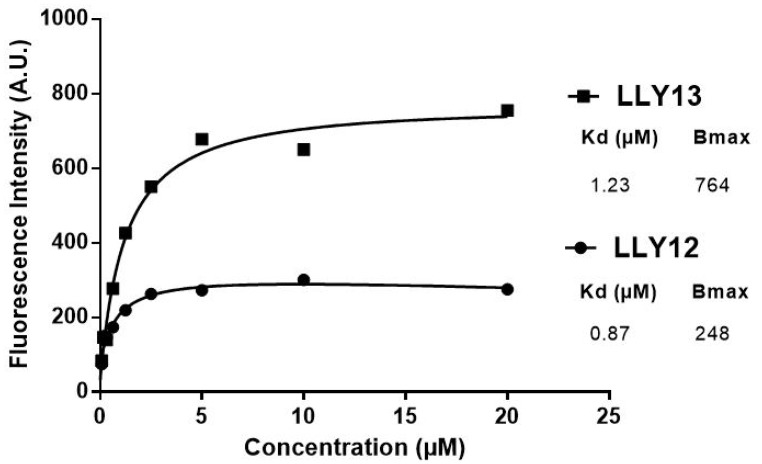
Comparison of binding affinity of LLY12 and LLY13. Flow cytometry analysis indicated that LLY13 has much higher maximum binding (Bmax 764 AU) compared to LLY12 (Bmax 248 AU), although Kd of LLY13 and LLY12 are comparable, 1.23 μM vs 0.87 μM respectively.

**Figure 5:**
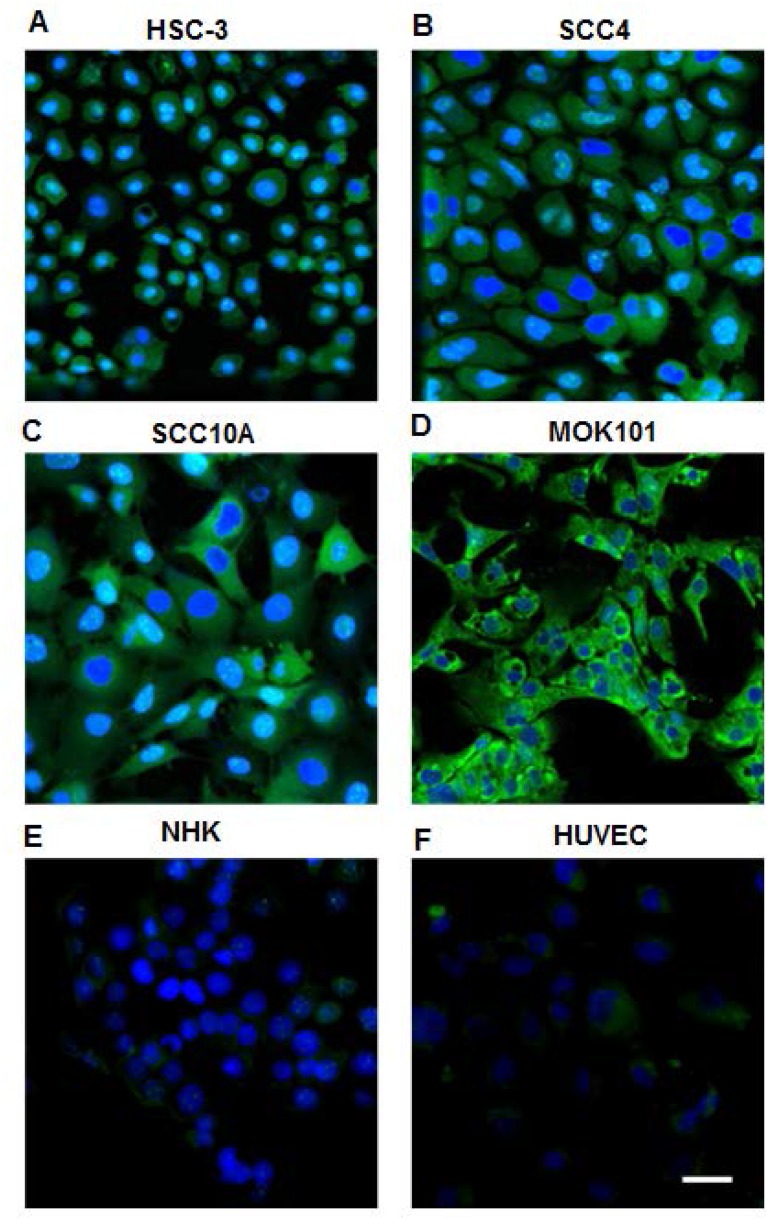
Confocal images of OSC cell lines and normal human cells stained by biotinylated LLY13/streptavidin-Alexa488. 1 μM of biotinylated LLY13 was added to cells grown in chamber slides and incubation for 30 minutes, followed by incubation of 1:500 streptavidin-Alexa488 for 30 min; nuclei were counter stained with DAPI. There was strong staining of OSC cells (**A**–**D**: HSC-3, SCC-4, SCC-10A, and MOK-101 cells), but weak or background staining of NHK (**E**) and human cells HUVEC (**F**) with LLY13 fluorescent probe; Scale bar: 50 μm

Biotinylated LLY12 and LLY13 were synthesized and purified. Their purities were determined to be >95% by HPLC. Their chemical identities were confirmed by mass spectrometry (Figures 2, 3). As shown in Figure 4, flow cytometry analysis of cells treated sequentially with biotin-peptide followed by streptavidin-PE indicated that LLY13 has around 3-fold maximum binding capacity in comparison to LLY12 (Bmax value 764 for LLY13 vs. 248 for LLY12), but has a comparable Kd value with LLY12 (1.27 vs 0.87 mM). Therefore, LLY13 demonstrated stronger binding affinity than LLY12. As shown in Figure 5, strong fluorescent signal was seen with MOK101, followed by HSC-3, SCC-4 and SCC10a oral cancer cells, after sequential treatment with 1 μM biotinylated LLY12 (biotin-LLY12) or biotinylated LLY13 (biotin-LLY13), followed by streptavidin-Alexa 488. Only weak background binding signals were observed for human keratinocytes and human endothelial cells.

### Assessment of LLY13 induced cancer cellular uptake phenomena

**Figure 6:**
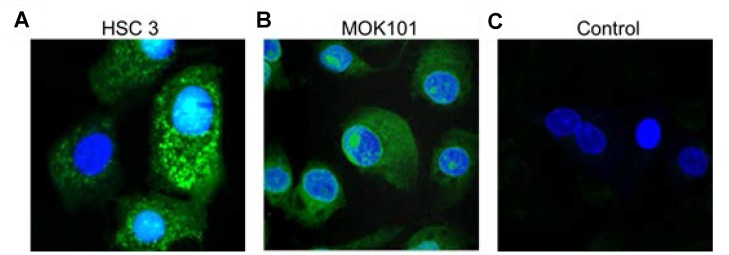
Confocal images of LLY13 peptide-dye conjugates taken up by live cells in culture. LLY13/streptavidin-488 complex were uptaken by oral cancer cells HSC-3 cells (**A**) and MOK-101 cells (**B**) after 3 hours of incubation. No signal was observed in control group using bacterial binding peptide/strepavidin-488 complex (**C**). Scale bar: 10 μm.

In order to dynamically observe the LLY13 induced cellular uptake by OSC cells, pre-mixed biotin-LLY13/streptavidin-Alexa488 complex was added to HSC-3 cells and MOK-101 cells. Cellular uptake of biotin-LLY13/streptavidin-Alexa488 complex was examined at time of 1, 2 and 3 hours. As shown in Figure 6, significant accumulation of LLY13 peptide-dye conjugates was seen in both HSC-3 and MOK-101 cytoplasm and in the nucleus; while there was no fluorescent signal observed in the control group, using an unrelated biotinylated pentapeptide that binds to intact bacteria.

### *In viv*o study of targeting efficiency of LLY13

**Figure 7:**
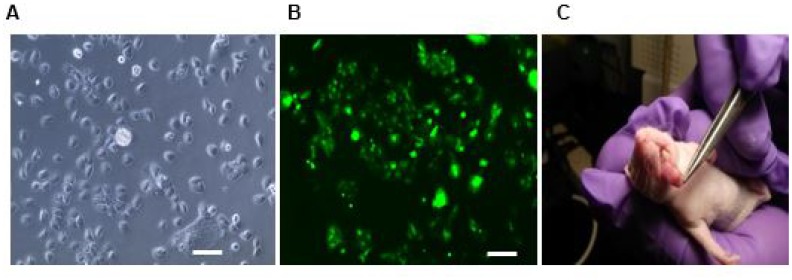
Luciferase/GFP transfected HSC3 cell implanted orthotopic mice tumor. (**A**) Light microscopy of transfected HSC-3 cells. (**B**) Fluorescent microscopy of luciferase/GFP transfected HSC-3 cells. (**C**) Photograph of orthotopically implanted HSC-3 cells into the tongue. Scale bar: 100μm.

**Figure 8:**
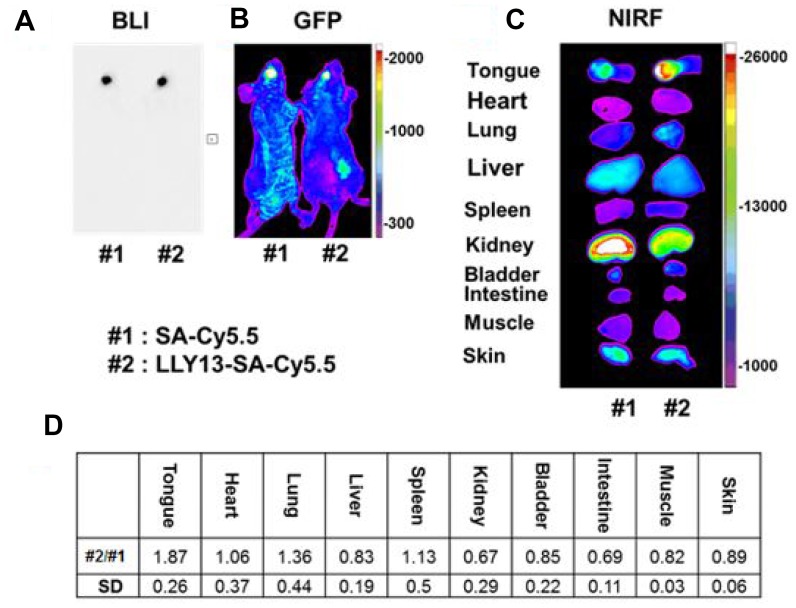
*In vivo* and *ex vivo* bioluminescence (BLI) and optical NIRF imaging. Images were taken 24 hours after tail vein injection of LLY13-biotin/streptavidin-Cy5.5. There was an overlap between tumor bioluminescence signal (**A**) and GFP signal (**B**). Compared to the control group with biotin/streptavidin-Cy5.5, LLY13 signal was 1.87 fold higher within the orthotropic implanted tumor in the tongue (**C** & **D**), p <0.05 (n=3).

**Figure 9:**
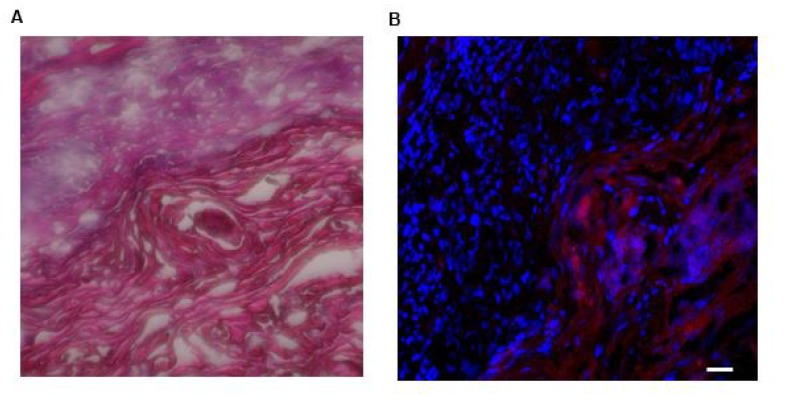
Photomicrographs of H&E staining of and LLY13-biotin/SA-Cy5.5 uptake into excised HSC3 implanted tongue tissues (20X). (**A**) H&E staining of excised HSC-3 bearing tongue. (**B**) LLY13-biotin/streptavidin-Cy5.5 localized within the tumor tissue as well as clearly demarcated the HSC-3 tumor (red) margins. Scale bar: 50μm.

*In vivo* tumor-targeting efficiency of LLY13 was evaluated in orthotopic GFP/luciferase transfected xenograft tumor of HSC-3 cells in mice with optical imaging. Briefly, HSC-3 cells were transfected with luciferase and GFP reporter plasmid. The GFP/luciferase transfected HSC-3 cells were implanted into the tongue to generate orthotropic luciferase transfected xenograft tumor (Figure 7). After tumor growth to 2.5 × 2.5 × 2.5 mm, pre-mixed biotin-LLY13/streptavidin-Cy5.5 complex was injected via tail vein. The mice were subjected to both *in vivo* and *ex vivo* bioluminescent imaging and optical near infra-red (NIRF) imaging. There were co-localization or overlapping signals between the bioluminescent image and optical NIRF image in the orthotopic xenograft tumors. We quantified the NIRF images and found that the orthotopic xenografts had significantly higher uptakes of biotin-LLY13/SA-Cy5.5 compared to that of biotin/streptavidin-Cy5.5 control (1.87-fold) (Figure 8). H&E staining of excised HSC-3 bearing tongue clearly demonstrated that the biotin-LLY13/streptavidin-Cy5.5 complex localized within the tumor tissue and clearly demarcated the HSC-3 tumor margins (Figure 9).

### Investigation of the receptor involved for LLY13

LLY12 and LLY13 have sequence homology with our previously reported α3β1 integrin targeting ligands [22–25]. We suspect these peptides might also target the same integrin. Using flow cytometry with fluorescent labeled anti-integrin antibodies, we observed that HSC-3 cancer cells did express α3-integrin (data not shown). In the bead binding-blocking assay, a panel of anti-integrin monoclonal antibodies (anti-α1, α2, α3, α4, α5, α6, αV, and β1) was each separately incubated with HSC-3 cancer cells prior to addition of LLY13 peptide-beads. Marked inhibition of cell binding to LLY13 peptide beads was observed only with anti-α3 integrin antibody blocked cells. Furthermore, the parent K562 cell, which has no α3 integrin transfected, showed no binding to LLY13-bead, whereas α3 integrin transfected K562 cells demonstrate moderate binding to LLY13-bead. Together, these results indicate that LLY13 target α3 integrin, which is over expressed in HSC-3.

## DISCUSSION

Optical imaging is useful as a non-invasive preclinical imaging tool to evaluate or screen tumor-targeting ligands [26, 27]. Optical probes are simpler to develop technically, non-radioactive and relatively inexpensive. In this report, we select two OSC targeting probes LLY12 and LLY13 identified from screening OBOC libraries for further biological evaluation. In flow cytometry analysis, LLY13 possessed higher binding affinity compared to LLY12. Therefore, LLY13 was selected as the lead peptide for further studies. In the staining assay with various OSC cells grown on chamber slides, LLY13 was able to detect OSC cells at 1 μM, including HSC-3, SCC-4, SCC-10a and MOK-101, while there were weak or background binding signal on the normal human keratinocyte and endothelial cells.

During the peptide-histochemical studies with OSC probes, we noticed the presence of some fluorescent signal inside live OSC cells after LLY13 was added, which indicates that LLY13 is able to penetrate into the cancer cells via endocytosis. To confirm this phenomenon, we added pre-mixed biotin-LLY13/streptavidin-Alexa488 complexes at different time intervals (30 min, 1 h, 2 h, and 3 h) to HSC-3 cells and MOK-101 cells grown on the chamber slides. Under confocal microscope examination, we observed that the LLY13 peptide-dye conjugates accumulated both in the cytoplasm and in the nucleus over time. In contrast, no obvious fluorescent signal was detected in the control group, using a bacterial binding peptide (biotin-peptide/streptavidin-Alexa488). The peptide induced cell internalization phenomenon was not observed with the peptide LLY12. These observations indicated that LLY13 not only targeted the surface of tumor cells but was also able to internalize inside the target cells. This OSC cancer cell penetrating peptide might be exploited as a vehicle to facilitate the delivery of nanocarriers to and inside the target tumor cells, where the drug will be released.

To evaluate the potential of LLY13 as a vehicle for anti-cancer drug delivery against OSC, we have established subcutaneous and orthotopic OSC xenograft models, and used *in vivo* and *ex vivo* NIR optical imaging to track the biodistribution of LLY13 in these animals. NIR probe exhibits modest tissue penetration (~1 mm) and minimal auto-fluorescence. Cy5.5 has been used previously by several groups as a convenient NIR fluorescent dye for *in vivo* detection of tumors [28, 29]. After given intravenously approximately 7.2 nmol OSC probe complex (biotin-LLY13/streptavidin-Cy5.5) to the mice, tumor uptake of the peptide dye conjugates was found to peak at about 24 hours post-injection followed by slow clearance; significant fluorescent signal remained at the tumor site even after 72 hours. The LLY13 targeting efficacy and specificity were further confirmed by H&E staining. As shown in Figure 9, LLY13 probe was found to be localized within the tumor tissue and not surrounding normal tissues, thus clearly demarcating the HSC-3 tumor margins. It has been observed that there were some Cy5.5 uptake in normal organs such as liver, skin and kidney etc., which partly was induced by the nonspecific binding from streptavidin-Cy5.5, demonstrated by sample #1 in Figure 8. We will in future studies directly label LLY13 with NIRF dye or radionuclide, without the use of streptavidin, for *in vivo* biodistribution studies.

To investigate the targeted receptor of LLY13, we employed an integrin antibody blocking assay [30, 31]. After HSC-3 oral cancer cells were treated separately with a panel of anti-integrin monoclonal antibodies (anti-α1, α2, α3, α4, α5, α6, αV, β1) prior to incubation with LLY13-beads, we found that only anti-α3 antibody could partially block HSC-3 cancer cell binding to LLY13-beads, indicating that the receptor for LLY13 might be α3 integrin. Molecular interactions between LLY13 and α3 integrin were further elucidated using K562 myeloid leukemia cells transfected with mutant α3-integrins. K562 cells transfected with α3 integrin and parent K562 cells were incubated with LLY13 compound beads. The α3-transfected K562 cells exhibited moderate binding to LLY13 compound beads, whereas K562 parent cells did not. This further demonstrated the binding specificity of LLY13 to α3-integrin on HSC-3 cells. Integrin exists as obligated heterodimer on cell membrane and α3 chain commonly pair with β1 chain of integrin. The inability of antibody against β1 chain to block HSC-3 cell binding to LLY13-beads suggests that the antibody interacts with the β1 chain outside of the binding pocket for LLY13.

Work is currently underway in our laboratory to evaluate the binding profile of LLY13 to cancer tissues obtained directly from primary human OSC tumors as well as a series of pre-cancerous lesions (such as leukoplakia, erythroplakia, or combined pathologies). Meanwhile, we are exploiting the use of LLY13 as a selective targeting agent for delivery of imaging probe and nanotherapeutic agent against OSC, similar to other targeting ligands developed in the Lam laboratory [28, 32, 33]. We expect that LLY13 will further enhance the therapeutic efficacy of the recently reported photo-responsive micellar nanoparticles against OSC xenografts in athymic mice [34, 35]. Finally, as LLY13 is cyclic and contains some D-amino acids and unnatural amino acids, it is expected to resist proteolytic degradation and be stable for *in vivo* application. Therefore, LLY13 is an excellent ligand that has great translational potential in tumor-specific imaging and chemotherapeutic drug delivery to the tumor site while sparing normal tissues. If needed, LLY13 can be further optimized using highly focused OBOC combinatorial libraries based on the structural motif of LLY13. These focused libraries can then be screened under higher stringency by lowering the bead surface substitution, shortening the incubation time or by adding soluble competing receptor specific antagonist against OSC, such that peptides with higher affinity and higher specificity against OSC might be identified.

## MATERIALS AND METHODS

### Cell lines

Oral cancer cell lines of OSC HSC-3, SCC-4, and normal human cells of NHK, HUVEC, were purchased from American Type Culture Collection, except as otherwise described. OSC cells of SCC-10a and MOK-101 were the gifted from Department of Orofacial Sciences, Pathology and Radiation Oncology, University of California, San Francisco.

### Synthesis of biotinylated LLY12 and LLY13

Peptide-biotin was designed to have biotin attached to the side chain of Lys, and two hydrophilic linkers between peptide and Lys (biotin). The synthesis employed a standard solid-phase peptide synthesis approach using fluorenylmethyloxycarbonyl (Fmoc) chemistry and 6-Chloro-1-hydroxybenzotriazole (6-Cl HOBt)/1,3-Diisopropylcarbodiimide (DIC) coupling as described in our previous publications [17, 25]. Rink amide MBHA resin (loading 0.503 mmol/g, P3 BioSystems, Louisville, KY) was used as solid support. In brief, a 4-fold molar excess of Fmoc-protected amino acids to resin was used for coupling. The reaction was monitored with the ninhydrin test. The Fmoc group was de-protected with 20% 4-methylpiperidine in *N*,*N*-dimethylformamide (DMF) (first 5 min, then 15 min). After the last cycle of amino acids coupling and Fmoc-deprotection, the linear biotinylated peptide was cleaved with trifluoroacetic acid (TFA) cocktail containing 82.5% TFA, 5% phenol, 5% thioanisole, 5% H_2_O and 2.5% triisopropylsilane. The liquid was collected and precipitated with cold diethyl ether. After 3 times of washing with ethyl ether, the precipitate was re-dissolved in 50% 0.1 M ammonium acetate buffer in acetonitrile (ACN) and cyclized with CLEAR-OX resin (Peptide International Inc, Louisville, KY) at room temperature for 2–4 h. Ellman test was negative to confirm cyclization completion. The liquid was collected by filtration. The beads were washed with a small amount of 50% ACN/water. The combined solution was lyophilized to obtain powder. The powder was re-dissolved in small amount of 50% ACN/water, and then purified by reversed-phase high performance liquid chromatography (RP-HPLC) on a preparative Vydac C18 column. The purity was determined to be >95% (Figure 2B and 3B). The identities of peptides were confirmed with orbitrap electrospray ionization mass spectrometry (ESI MS) (Figure 2C and 3C).

### Cytotoxicity evaluation of identified OSC-binding peptides

MTS assay was used to evaluate the potential cytotoxicity of peptides against normal human keratinocytes [36, 37]. Briefly, 2 × 10^3^ NHK cells were seeded in 96 well tissue culture plates and analysis was performed 48 hours after treatment with LLY12 and LLY13 by adding 20 μL per well of the tetrazolium salt MTS, 3-(4,5-dimethylthiazol-2-yl)-5-(3-carboxymethoxyphenyl)-2-(4-sulfophenyl)-2H-tetrazolium for 2 hours at 37° C. The 96 well plates were read on a VERSA max tunable microplate reader at 490 nm. Similar to the MTT assay, the reduction of MTS solutions to formazan reflects mitochondrial activity and cell viability by proxy.

### Flow cytometry study of binding affinity of ligands

Flow cytometry was employed to compare the binging affinity of LLY12 and LLY13 [25–27]. Confluent tumor cells (80–90%) were dissociated with 0.05% trypsin-EDTA and neutralized with culture medium. After washed three times with F10 buffer, containing PBS with 10% FBS and 1 mM MnCl_2_ , the human oral cancer cells HSC-3 (3 × 10^5^) were added with different concentration of biotinylated LLY12 and LLY13 in 50 μL F10 media for 30 minutes on ice. Then each sample was washed three times with 1 mL PBS containing 1% FBS. Samples thereafter were incubated with a 1:500 dilution of streptavidin-phycoerythrin (1 mg/mL) for 30 min on ice followed by a single wash with 1 mL of PBS containing 1% FBS. Finally, the samples in PBS were analyzed by flow cytometry and the mean fluorescence intensity (MFI) was acquired for each individual sample.

### Peptide-histochemical study of OSC-binding peptides

Peptide-histochemical assay was used as an *in vitro* study to evaluate the binding efficacy and specificity of LLY12 and LLY13 on various human OSC cell lines as well as normal human cells [38, 39]. Biotinylated LLY12 and LLY13 were prepared by adding a lysine (biotin) at the C-terminal of the ligands via two units of hydrophilic linker (AEEA) to form ligand-linker-lysine (biotin) (Figures 2, 3). The biotinylated peptides were synthesized on Rink amide AM resin using standard solid phase peptide synthesis approach and cyclized in solution using CLEAR-OX resin as we previously described [26]. Human OSC cells and normal human keratinocytes and endothelial cells were seeded on chamber slides. At 70% confluence, OSC cells were incubated with 5% BSA to block non-specific binding. Botinylated LLY12 or biotinylated LLY13 at different concentrations were then added to the OSC cells grown in the chamber slides and incubated for half an hour. After washing, 1:500 dilution of streptavidin-Alexa 488 was added and incubated for half hour. Specimens were then washed 3× with PBS and fixed briefly with 4% formaldehyde before adding the DAPI. Confocal microscopy was performed to observe the cell binding efficacy by the OSC targeting peptides.

### *In vitro* cellular uptake studies for LLY13

Peptide induced cell uptake assay was used to investigate endocytic uptake of the targeting peptide by live OSC cells [29, 40, 41]. Biotinylated LLY13 was pre-incubated (50 μM) with streptavidin-Alexa488 (1mg/mL) for 2 hours at room temperature and then at 4° C overnight to form the biotin-peptides/streptavidin-Alexa488 complex. Oral cancer cells of HSC-3 cells and MOK-101 were seeded in chamber slides at a concentration of 2.3 × 10^4^ cells. After 60% confluency, biotin-peptides-streptavidin-Alexa488 complexes were added at different time intervals (30 min, one hour, two hours and three hours). By the end of experiments, 1:2000 dilution of DAPI was added to the specimens. Peptide induced endocytic uptake of cancer cells was observed under confocal laser scanning microscope.

### Establishment of subcutaneous and orthotopic mice xenografts

Subcutaneous and orthotopic luciferase-transfected OSC xenograft models were used to evaluate ligands’ targeting efficacy [42]. All animal experiments were performed in compliance with the institutional guidelines according to protocol No. 07-13119 and No. 09-15584 approved by the Animal Use and Care Administrative Advisory Committee at the University of California, Davis. Athymic nude mice (*nu*/*nu*) were obtained from Harlan (Hayward, CA, USA) at 5 to 6 weeks of age. OSC subcutaneous tumors were established by injecting HSC-3 (1 × 10^6^ cells) into the right flank (*n* = 20) [28, 30, 31]. To establish OSC orthotopic bioluminescent nude mice model in tongue, HSC-3 cells were transfected with lentiviral vectors that express green fluorescent protein (GFP)/firefly luciferase fusion proteins. Twenty μL of 1.2 × 10^4^ of infected HSC-3 cells mixed with Matrigel at 1:1 were implanted into mouse tongues. Tumors were measured with calipers 2 times per week and the volume was calculated by the formula for hemiellipsoids [17, 25].

### NIRF optical imaging

*In vivo* and *ex vivo* optical imaging studies were performed to evaluate the bio-distribution and targeting efficacy [25]. OSC subcutaneous and orthotopic mice models were established as described above. When the orthotopic tumors in the tongue and the subcutaneous tumor in the flank reached approximately 2.5 × 2.5 × 2.5 mm^3^ and 6 × 6 × 6 mm^3^, Cy5.5 labeled tetravalent OSC probe, prepared by mixing 7.2 nmol of biotinylated peptide with 1.8 nmol of streptavidin-Cy5.5 in PBS overnight at 4°C, was injected into the mice via the tail vein. Twenty-four hours after injection, the mice were anesthetized by an injection of 30 mL Nembutal (50 mg/mL) and images were acquired with a Kodak IS2000MM Image Station with excitation filter 625/20 band pass, emission filter 700/35 band pass, and 150 W quartz halogen lamp light source set at maximum. The mice were then sacrificed, and the organs were excised for *ex vivo* imaging. Data were collected and analyzed using the Kodak ID 3.6 software by mapping the region of interest on the images.

### Histological study

Hematoxylin and eosin (H&E) staining of excised OSC bearing tongues were performed to evaluate the targeting efficacy [38, 39]. Three mice with OSC tumors implanted in the tongue were randomly selected from each group and sacrificed with cervical dislocation. The tumor specimens from mice tongues were excised by cutting the tongue root and bathed in a neutral buffered 10% concentrated formalin solution and embedded for H&E staining. Histological assessment was conducted using optical microscopy.

### Investigation of corresponding receptors

Antibody-blocking assay was used to investigate the corresponding receptor involved [30, 31]. Briefly, OSC cells were harvested by trypsinization, washed once in phosphate-buffered saline and re-suspended in F10 buffer (1× PBS, 10% FBS, 0.1% NaN_3_, 1 mm CaCl_2_, and 1mm MgCl_2_). The OSC cells were then incubated with integrin blocking kit (containing α1, α2, α3, α4, α5, α6, αV, and β1 antibody) at 1 μg per 1million cells for two hours. Integrin blocked OSC cells were washed with PBS 3× and then incubated with LLY13-beads for two hours. The cell binding blocking effects of these peptide beads were observed under inverted microscope.

An additional assay was carried out by using specific α3 integrin-transfected K562 cells in order to confirm the hypothesized involvement of α3 receptor in LLY13 peptide binding. Briefly, LLY13 peptide beads were incubated with α3-transfected K562 cells as well as K562 cells for three hours. The effect of cell binding to LLY13 peptide beads was observed under inverted microscope.

### Statistical analysis

Statistical analysis was performed by Student’s *t*-test for two groups, and one-way analysis of variance for multiple groups. All results were expressed as the mean ± std. unless otherwise noted. *P value* < 0.05 was considered statistically significant.

## CONCLUSION

We identified specific cell surface targeting peptides against OSC through combinatorial OBOC libraries. *In vitro* studies show that LLY12 and LLY13 are able to detect different OSC cells at the concentration of 1 mM after 30 minutes incubation. *In vitro* studies demonstrated that LLY13 is capable of inducing OSC cell endocytosis, and accumulation both in cytoplasm and nucleus. *In vivo* studies also demonstrate that LLY13 is able to accumulate in the OSC tumors and demarcate the tumor margins in orthotopic xenograft model in athymic mice. Therefore, LLY13 represents a promising candidate for further study in the domain of targeted tumor imaging as well as anti-tumor targeting therapy against OSC.
